# COVID-19 vaccine acceptance and perceived stigma in patients with depression: a network perspective

**DOI:** 10.1038/s41398-022-02170-y

**Published:** 2022-10-04

**Authors:** Hong Cai, Wei Bai, Xiangdong Du, Ling Zhang, Lan Zhang, Yu-Chen Li, Huan-Zhong Liu, Yi-Lang Tang, Todd Jackson, Teris Cheung, Feng-Rong An, Yu-Tao Xiang

**Affiliations:** 1 Unit of Psychiatry, Department of Public Health and Medicinal Administration, & Institute of Translational Medicine, Faculty of Health Sciences, University of Macau, Macao, Macao SAR China; 2 Centre for Cognitive and Brain Sciences, University of Macau, Macao, Macao SAR China; 3 Institute of Advanced Studies in Humanities and Social Sciences, University of Macau, Macao, Macao SAR China; 4 Guangji Hospital Affiliated to Soochow University, Suzhou, Jiangsu province China; 5 Nanning Fifth People’s Hospital, Nanning, Guangxi province China; 6 Department of Psychiatry, Lanzhou University Second Hospital, Lanzhou, Gansu province China; 7 Department of Psychiatry, Xiamen Xianyue Hospital, Xiamen, China; 8 Department of Psychiatry, Chaohu Hospital, Anhui Medical University, Hefei, China; 9 School of Mental Health and Psychological Sciences, Anhui Medical University, Hefei, China; 10 Department of Psychiatry and Behavioral Sciences, Emory University, Atlanta, GA USA; 11 Atlanta VA Medical Center, Atlanta, GA USA; 12 Department of Psychology, University of Macau, Macao, Macao SAR China; 13 School of Nursing, Hong Kong Polytechnic University, Hong Kong, Hong Kong SAR China; 14 The National Clinical Research Center for Mental Disorders & Beijing Key Laboratory of Mental Disorders Beijing Anding Hospital & the Advanced Innovation Center for Human Brain Protection, Capital Medical University, Beijing, China

**Keywords:** Depression, Scientific community

## Abstract

The association between coronavirus disease (COVID-19) vaccine acceptance and perceived stigma of having a mental illness is not clear. This study examined the association between COVID-19 vaccine acceptance and perceived stigma among patients with recurrent depressive disorder (depression hereafter) using network analysis. Participants were 1149 depressed patients (842 men, 307 women) who completed survey measures of perceived stigma and COVID-19 vaccine attitudes. T-tests, chi-square tests, and Kruskal–Wallis tests were used to compare differences in demographic and clinical characteristics between depressed patients who indented to accepted vaccines and those who were hesitant. Hierarchical multiple regression analyses assessed the unique association between COVID-19 vaccine acceptance and perceived stigma, independent of depression severity. Network analysis examined item-level relations between COVID-19 vaccine acceptance and perceived stigma after controlling for depressive symptoms. Altogether, 617 depressed patients (53.7%, 95 confidence intervals (CI) %: 50.82–56.58%) reported they would accept future COVID-19 vaccination. Hierarchical multiple regression analyses indicated higher perceived stigma scores predicted lower levels of COVID-19 vaccination acceptance (*β* = −0.125, *P* < 0.001), even after controlling for depression severity. In the network model of COVID-19 vaccination acceptance and perceived stigma nodes, “Feel others avoid me because of my illness”, “Feel useless”, and “Feel less competent than I did before” were the most influential symptoms. Furthermore, “COVID-19 vaccination acceptance” had the strongest connections with illness stigma items reflecting social rejection or social isolation concerns (“Employers/co-workers have discriminated”, “Treated with less respect than usual”, “Sense of being unequal in my relationships with others”). Given that a substantial proportion of depressed patients reported hesitancy with accepting COVID-19 vaccines and experiences of mental illness stigma related to social rejection and social isolation, providers working with this group should provide interventions to reduce stigma concerns toward addressing reluctance in receiving COVID-19 vaccines.

## Introduction

Recurrent depressive disorder (depression hereafter) is among the most prevalent mental disorders, affecting approximately 5–6% of people worldwide [1]. Since coronavirus disease (COVID-19) was first reported in early 2020, it has emerged in to more than 200 countries [2]. Psychiatric disorders are associated with an increased risk of COVID-19 infections in addition to higher hospitalization, morbidity, and mortality rates due to its variants [3, 4]. For example, compared with patients who do not have psychiatric disorders, patients with severe psychiatric disorders, including depression, have a higher risk of COVID-19 mortality (odds ratio (OR): 2.26; 95% confidence interval (CI): 1.18–4.31) [4]. Depressed patients have increased risk for COVID-19 infection and severe health outcomes for several reasons. First, depression is associated with altered immune function involving a pro-inflammatory state and maladaptive T-cell functioning [5–9]. Second, depressed patients often suffer from sleep disturbances, which are associated, in turn, with dysregulated immune system functioning and increased risk of infection [10–13]. Third, some depressed patients do not have healthcare insurance coverage and cannot receive timely treatment when necessary [13–16]. Finally, due to impairments in cognitive and social functioning, some depressed patients may have difficulty complying strictly with preventive measures against COVID-19.

As a major component in the fight to control the COVID-19 pandemic, many countries have initiated COVID-19 vaccination programs for eligible individuals. Most currently approved COVID-19 vaccines require at least two doses, though effectiveness durations of vaccines are not yet clear [17, 18]. Barriers to receiving adequate healthcare, including vaccinations, are also significant for patients with severe mental illnesses [19]. Aside from more limited access to public health recommendations such as facial masking and social distancing and poor adherence to these measures [20], the stigma of having a mental illness and discrimination are potentially important barriers to seeking healthcare among people with psychiatric disorders [21, 22]. Stigma refers to extreme disapproval of a person or group based on characteristics that distinguish them from other members of society, including skin color, sexual orientation or presence of a mental illness [23]. Stigmatization occurs simultaneously at intrapersonal (e.g., self-stigma), interpersonal (e.g., relations with others), and structural (e.g., discriminatory and/or exclusionary policies, laws, and systems) levels [24]. People with a history of depression or other mental disorders report that messages they receive about their illness can make them feel labeled, judged, devalued, dismissed, and dehumanized by others, including health professionals with whom they come into contact [25–30]. Stigma experiences and internalization of such disapproval may fuel depression and shame about having a mental illness, limit social interactions and contribute to inadequate healthcare [24], in part, because patients are concerned about being judged or discriminated against or because they perceive the health care system as a threat for stereotyping them [30, 31].

Recent reviews of the literature have confirmed that stigmatizing experiences and discrimination are associated with increased reluctance to seek healthcare among psychiatric patients. For example, in a review of 123 articles on stigma among psychiatric patients in Asian countries, Zhang et al. [32] concluded that patients receive inadequate care, in part, because they are viewed as dangerous and psychiatric illnesses in Asian societies are less socially-acceptable (i.e., seen as reflections of personal weaknesses). Although some studies have focused on associations between mental illness stigma and treatment of the mental illness itself, another review of 144 studies based on over 90,000 patients [33] found high levels perceived stigma related to having a mental illness were linked to increased reluctance to use primary or secondary/tertiary health care services [33].

To date, studies on the acceptability of COVID-19 vaccination have found moderate to high acceptance rates among adolescents and adults, respectively [34, 35]. Predictors of vaccine acceptance include perceived benefits and efficacy of the COVID-19 vaccine [36–38]. However, findings on vaccine acceptance in the general public are not necessarily applicable to patients with severe psychiatric disorders. Furthermore, studies have not evaluated links between perceptions of being stigmatized and acceptance of COVID-19 vaccines in patients with mental illnesses nor have specific COVID-19 vaccination guidelines been developed for these populations. In light of evidence that stigma, discrimination, and negative attitudes toward severe mental illnesses correlate with general reluctance to seek healthcare among affected individuals, examining links between the stigma of mental illnesses such as depression and attitudes toward specific interventions such as acceptance of COVID-19 vaccinations may aid in reducing negative consequences the virus has for such at-risk groups [3–5].

Past studies on links between perceived stigma of mental illness and health care use also tended to conceptualize stigma as a broad construct that could be assessed on the basis of summing item scores on questionnaires and the implicit assumption that individual experiences tapped by each item are equally important and interchangeable [26–28] A potential drawback of relying upon total or average stigma scale scores in this context is that information on associations between individual stigma items and health care use attitudes can be obscured [39].

Network analysis (NA), a widely used approach to mapping potential relationships among particular symptoms of psychiatric disorders, can address this limitation. Network analysis is designed to identify (1) central nodes or symptoms that are more likely to activate other symptoms and contribute to the onset and/or maintenance of a disorder or related problems as well as (2) “bridge” nodes that have the strongest links with comorbid disorders or co-occurring experiences [40]. As such, network analysis is particularly useful when investigating connections between different domains of interest (i.e., comorbid characteristics, risk factors, and symptoms) [41–43] and is uniquely suited for identifying specific symptoms or attributes that may be the most useful targets for treatment [44].

In sum, previous studies have established significant overall associations between feeling stigmatized for having a mental illness and reluctance to seek healthcare. However, it is not yet clear whether higher overall levels of reported stigma with having a mental illness or particular stigma experiences are associated with increased reluctance to seek specific pandemic-related interventions (i.e., COVID-19 vaccinations). The main purposes of this study were two-fold. First, we explored overall associations between stigma concerns related to having depression and COVID-19 vaccination acceptance among depressed patients. Second, we used network analysis to identify central nodes and bridge nodes within the network of item level associations between facets of perceived stigma and COVID-19 vaccination acceptance in this group.

## Methods

### Study design

This cross-sectional, observational study was conducted from October 1, 2020 to August 15, 2021 in six major psychiatric hospitals distributed in east, west, south and north regions of China (i.e., Beijing, Guangxi, Jiangsu, Fujian, Gansu and Anhui). Due to safety guidelines adopted during the COVID-19 pandemic, face-to-face assessments were not adopted. Following other studies [45, 46], the WeChat-based “QuestionnaireStar” program was used with a consecutive sampling method. WeChat is a widely used social communication application with more than 1.2 billion active users in China. All patients who attended outpatient clinics or received inpatient services in participating psychiatric hospitals during the study period were consecutively invited to volunteer. Patients were invited to scan a Quick Response code (QR Code) linked to the introduction and invitation of this study with their own or a guardian’s smartphone. After providing electronic written informed consent, patients could access the data collection form and questionnaire. To be eligible, participants met the following criteria: (1) aged 18 years or older; (2) diagnosed with recurrent depressive disorder based on the International Classification of Diseases, Tenth Revision (ICD-10) [47]; (3) able to read and understand Chinese. The study protocol was centrally approved by ethics committees of Beijing Anding Hospital and the other participating hospitals.

### Data collection and measures

Socio-demographic data collected using a pre-designed data collection sheet, included gender, age, place of residence (urban/rural), marital status (married/unmarried), living status (living alone/living with family or friends or others), education level (high school and below/college education and above), perceived health status (poor/fair/good) and perceived economic status (poor/fair/good). Following a previous study on influenza vaccine attitudes [48], one standardized question was added to measure COVID-19 vaccine acceptance: “Do you intend to be vaccinated against COVID-19 in the future?” There were three response options (e.g., “I would be vaccinated against COVID-19”, “I would not accept COVID-19 vaccination temporarily”, and “I would refuse to accept a COVID-19 vaccination”), the latter two of which were collapsed into “do not accept COVID-19 vaccination”.

Severity of depressive symptoms was measured using the validated Chinese version of the two item-Patient Health Questionnaire (PHQ-2) [49, 50]. Total PHQ-2 scores ranged from 0 to 6, with higher scores representing more severe depressive symptoms. The reliability and validity of the Chinese version have been supported in past research [50]. Suicidality was assessed with a single item (“Over the past year, have you thought that you would be better off dead or made a plan or attempt for suicide?”) that included a binary response option (yes/no). Severity of fatigue was assessed using a fatigue numeric rating scale with options ranging from ‘0’ (no fatigue) to ‘10’ (extreme fatigue) [51]. Severity of physical pain was measured using a Visual Analog Scale for Pain (VAS) [52] with anchors of ‘0’ (no pain at all) and ‘10’ (worst pain imaginable). Reliability and validity of the VAS Chinese version have also been supported [53]. Experiences of the perceived stigma associated with having depression were measured with the 24-item Social Impact Scale (SIS) [54]. Each SIS item is rated from ‘1’ (strongly disagree) to ‘4’ (strongly agree), with total scores ranging from 24 to 96. Higher scores indicate more severe stigma. The SIS Chinese version has been validated with good psychometric properties [55].

### Statistical analysis

#### Univariate and multivariate analyses

Data analyses were performed using SPSS version 25.0 (SPSS Inc., Chicago, Illinois, USA). Distributions of all continuous variables were checked for normality using P-P plots. Chi-square tests, independent samples t-tests, and Mann–Whitney U tests were used, as appropriate, to compare depressed patients who would accept future COVID-19 vaccination versus those who would not accept future vaccination on sociodemographic factors, disease-related variables, and perceived stigma. To examine whether there was an independent association between perceived stigma and COVID-19 vaccination acceptance in the sample, a hierarchical multiple linear regression analysis was performed. COVID-19 vaccination acceptance was the dependent variable. In Block 1 of the regression model, suicidality and PHQ-2 total score were included as predictors in addition to any other measures that differed significantly between vaccine acceptance subgroups in univariate analyses. In block, SIS total score was entered as the predictor to examine whether perceived stigma made a unique, additional contribution to the prediction of COVID-19 vaccination acceptance, after the impact of all other significant predictors had been statistically controlled. Significant statistical differences were set at *P* < 0.05 (two-tailed).

#### Network structure

The network model was estimated using the R software [56]. We computed polychoric correlations between responses on the COVID-19 vaccination acceptance item and all SIS items to investigate edges of the network model, after controlling for depressive symptom severity. We also estimated the Graphical Gaussian Model (GGM), a popular network model, with the graphic least absolute shrinkage and selection operator (LASSO) and Extended Bayesian Information Criterion (EBIC) model using R package *‘qgraph’* [57]. GGM is a pairwise Markov random field (PMRF) model used for interval or ordinal data, in which edges are interpreted as partial correlation coefficients. The network was visualized using *‘qgraph’* package, where thicker edges represented stronger relations between nodes. We also estimated the centrality index, Expected Influence (EI) of nodes, to determine which SIS items were more central (influential) in the network model [58]. To identify SIS items that were directly associated with future COVID-19 vaccine acceptance, the “flow” function in R package ‘*qgraph’* was used [59]. In addition, to examine nodes that more often fell on the shortest predictive pathways from future COVID-19 vaccine acceptance to other nodes, we computed node-specific predictive betweenness as a centrality measure. Because betweenness is generally not a stable centrality measure [57], we used both nonparametric and case-drop bootstraps to investigate degree of variability in-betweenness [57]. Node-specific predictive betweenness of future COVID-19 vaccine acceptance (i.e., how often a node lies on pathways between two other nodes based on 1000 nonparametric bootstrap iterations, with “future COVID-19 vaccine acceptance” (COV) as one or the other node) was estimated in this study. The bootstrap method in *‘bootnet’* package investigated stability of the central index based on correlation stability coefficients (CS-coefficient). We set the CS-coefficient cut-off at 0.25 for all indices, because CS-coefficients are usually below 0.25 when centralities do not differ from one another [57].

## Results

### Participant characteristics

**Fig. 1:**
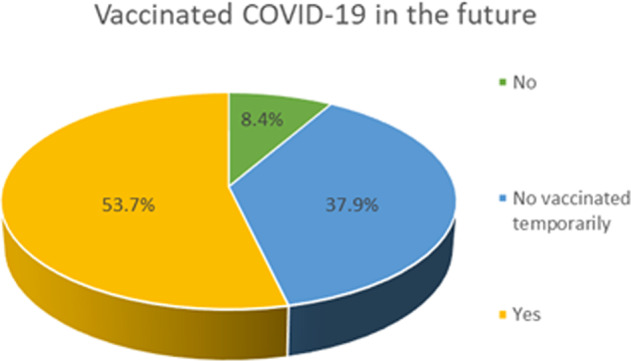
Behavior toward COVID-19 vaccines (*N* = 1149).

Of 1189 depressed patients invited to participate, 1149 completed the survey for a response rate of 96.6%. A total of 617 depressed patients (53.7%, 95% confidence intervals (CI): 50.82–56.58%) reported they would accept a future COVID-19 vaccination while 435 patients (37.9%, 95%CI: 35.10–40.66%) reported they would not accept COVID-19 vaccination temporarily, and 97 patients (8.4%, 95%CI: 6.8–10.1%) stated they would refuse to accept a COVID-19 vaccination (Fig. 1). Demographic and clinical characteristics of participants are shown in Table 1.

**Table 1:** Demographic characteristics of participants.

Variable	Total (*N* = 1149)		Acceptance of COVID-19 vaccination				*χ*^2^	df	*P*
			Acceptance (*N* = 617)		Hesitation (*N* = 532)				
	*N*	%	*N*	%	*N*	%			
Male gender	842	73.3	449	72.8	393	73.9	0.177	1	0.674
Married	489	42.6	276	44.7	213	40.0	2.576	1	0.109
College education and above	581	50.6	314	50.9	267	50.2	0.057	1	0.812
Living alone	113	9.8	60	9.7	53	10.0	0.018	1	0.893
Urban residence	803	69.9	441	71.5	362	68.0	1.597	1	0.206
Perceived health status							3.958	2	0.138
Poor	191	16.6	103	16.7	88	16.5			
Fair	779	67.8	406	65.8	373	70.1			
Good	179	15.6	108	17.5	71	13.3			
Perceived economic status							0.227	2	0.893
Poor	212	18.5	114	18.5	98	18.4			
Fair	846	73.6	452	73.3	394	74.1			
Good	91	7.9	51	8.3	40	7.5			
Having suicidality in the past year	782	68.1	400	64.8	382	71.8	6.393	1	**0.011**
Inpatients	175	15.2	121	19.6	54	10.2	19.804	1	**<0.001**
	**Mean**	**SD**	**Mean**	**SD**	**Mean**	**SD**	**t/Z**	**df**	***P***
Age (years)	30.76	14.34	30.93	14.31	30.56	14.37	−0.707	–^a^	0.480
Age of onset (years)	29.12	15.06	29.23	14.80	29.00	15.36	−0.740	–^a^	0.459
Fatigue total score	5.56	2.52	5.45	2.60	5.69	2.42	−1.493	–^a^	0.136
Physical pain total score	3.08	2.96	2.97	2.52	3.20	2.38	−1.996	–^a^	**0.046**
PHQ-2 total score	3.16	1.89	3.03	1.94	3.32	1.82	2.576	1147	**0.010**
SIS total score	57.91	11.52	56.19	10.80	59.39	11.92	−4.74	1147	**<0.001**

Bolded values: <0.05.

*M* mean, *SD* standard deviation, *COVID-19* Corona Virus Disease 2019, *PHQ-2* 2-item Patient Health Questionnaire, *SIS* Social Impact Scale.

^a^Mann–Whitney U test.

### Correlates of COVID-19 vaccination acceptance

Table 1 summarizes comparisons between depressed patients who would accept versus those who would not accept future COVID-19 vaccination on the main research measures. Those who accepted future vaccinations were significantly less likely to report suicidality in the past year (*p* = 0.011), more likely to report being inpatients (*p* < 0.001), and less likely to report severe physical pain (*p* = 0.046), or depression symptoms (*p* = 0.01). Depressed patients who accepted future COVID-19 vaccinations also reported significantly lower SIS scores (i.e., less perceived stigma related to having depression, *p* < 0.001). The hierarchical multiple regression analysis indicated total SIS scores had a significant inverse association with COVID-19 vaccination acceptance (*β* = −0.125, *R*^2^ = 0.013, *P* < 0.001), after statistically controlling for severity of depression, suicidality, and other significant correlates of vaccine acceptance.

### Structure of mental illness stigma-COVID-19 vaccine acceptance model

**Fig. 2:**
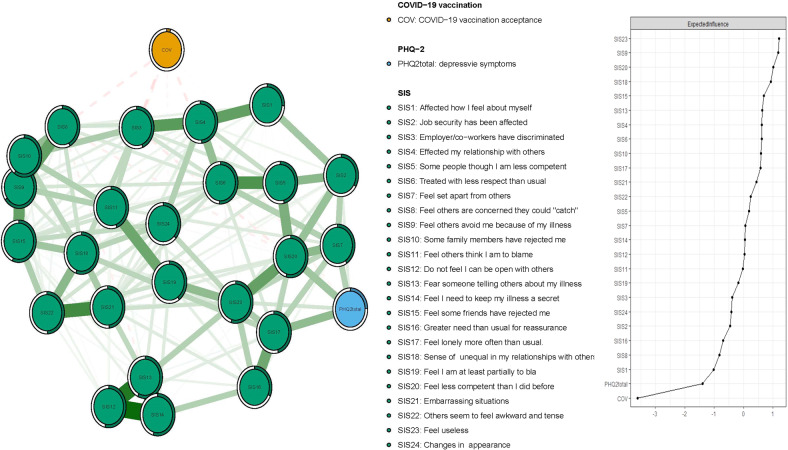
Network structure of COVID-19 vaccination acceptance and perceived stigma in depressed patients. Ring-shaped pie charts represent predictability (a fully filled dark ring would indicate that 100% of the symptom’s variance is explained by its inter correlations with the other symptoms in the network). In the diagram symptom nodes with stronger connections are closer to each other. The blue node denotes the PHQ-2 total score items (2-items Patients Health Questionnaire); the red node denotes the SIS items (Social Impact Scale). The dark green lines represent positive correlations. The edge thickness represents the strength of the association between symptom nodes.

Figure 2 presents the network structure of perceived illness stigma and COVID-19 vaccination acceptance, after controlling for depressive symptoms. The predictability of items is shown as ring-shaped pie charts in Fig. 2. The mean predictability of 0.46, indicated that, on average, 46% of the variance in each node could be accounted for by neighboring nodes. The model shows that the connection between nodes SIS9 (“Feel others avoid me because of my illness”) and SIS10 (“Some family members have rejected me”) (average edge weight = 0.37) was the strongest positive edge in the perceived stigma community, followed by edges between nodes SIS12 (“Do not feel I can be open with others about my illness”) and SIS14 (“Feel I need to keep my illness a secret”) (average edge weight = 0.35) and nodes SIS13 (“Fear someone telling others about my illness”) and nodes SIS14 (“Feel I need to keep my illness a secret”) (average edge weight = 0.33) (Supplementary Tables S1 and S2).

**Fig. 3:**
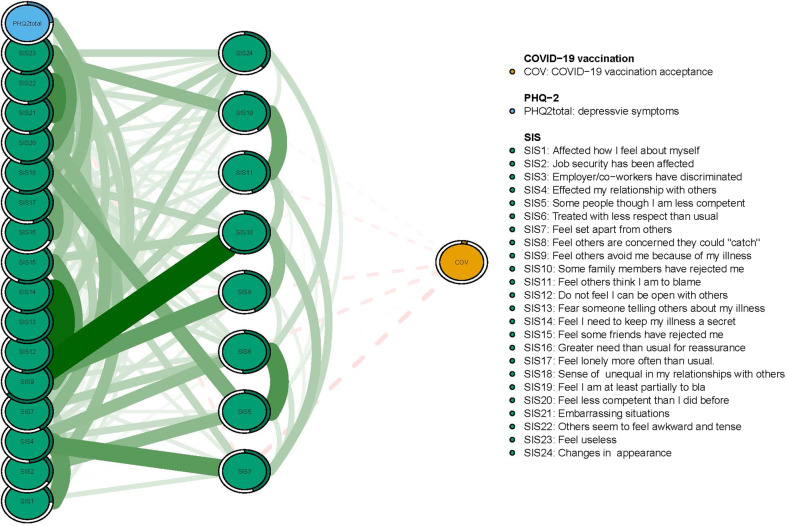
Flow network of future COVID-19 vaccine acceptance. Ring-shaped pie charts represent predictability (a fully filled dark ring would indicate that 100% of the symptom’s variance is explained by its inter correlations with the other symptoms in the network). In the diagram symptom nodes with stronger connections are closer to each other. The blue node denotes the PHQ-2 total score items (2-items Patients Health Questionnaire); the red node denotes the SIS items (Social Impact Scale). The dark green lines represent positive correlations. The red lines represent negative correlations. The edge thickness represents the strength of the association between symptom nodes.

**Fig. 4:**
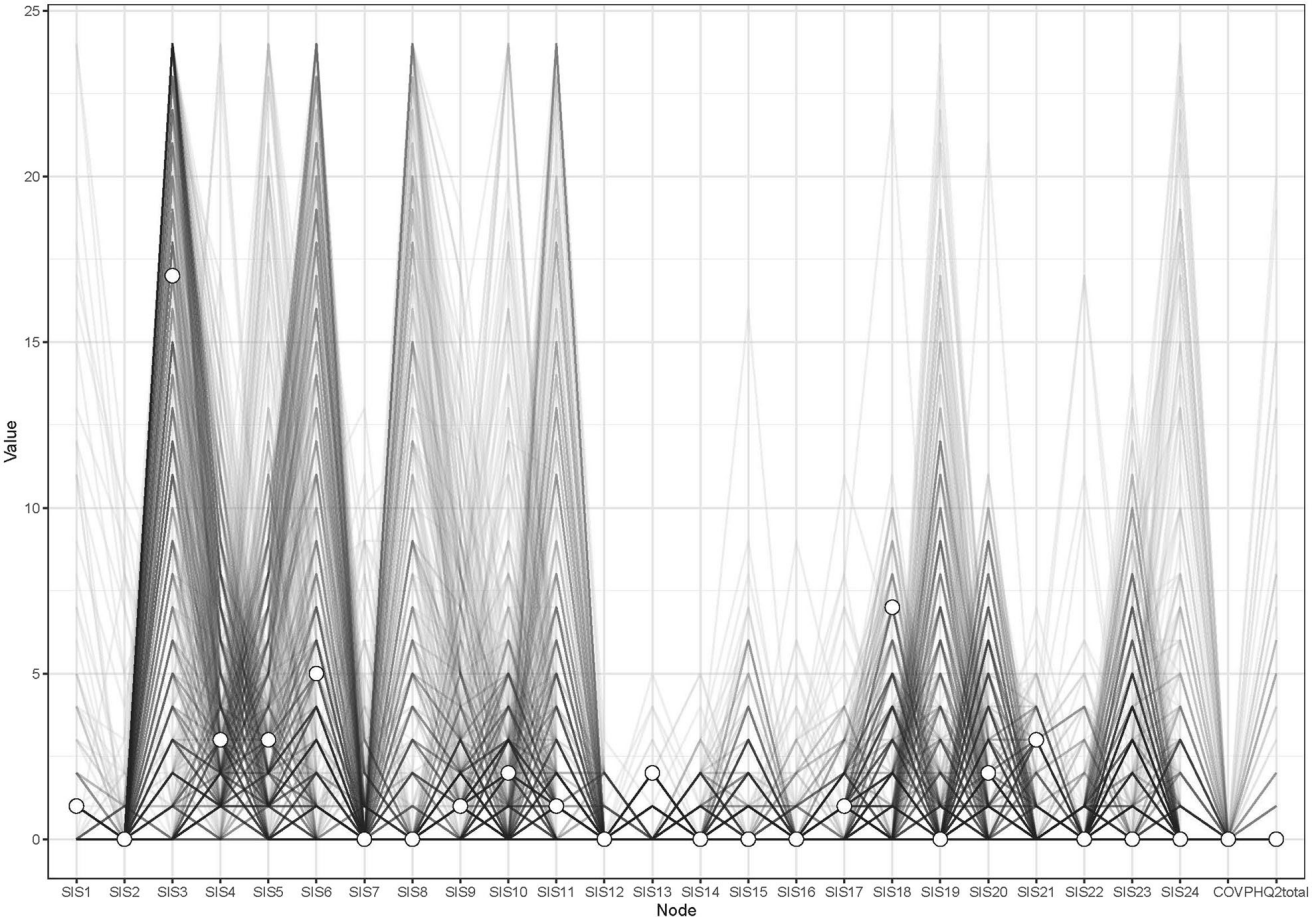
Node-specific predictive betweenness. The white dots represent the node-specific predictive betweenness in the study sample, while the black lines represent the variability of node-specific betweenness across 1000 nonparametric bootstrap iterations.

In terms of EI in the network model, the node SIS23 (“Feel useless”) had the highest EI centrality, followed by nodes SIS9 (“Feel others avoid me because of my illness”) and SIS20 (“Feel less competent than I did before”) (Fig. 2), hence indicating that these three symptoms were the most influential ones within the network model among depressed patients. In contrast, the item COV (“COVID-19 vaccination acceptance”) was the least central node (Fig. 2). Figures 3 and 4 show that SIS3 (“Employers/co-workers have discriminated”) (average edge weight = −0.046) was the strongest bridge node connecting to COV (“COVID-19 vaccination”), followed by SIS18 (“Sense of inequality in my relationships with others”) and SIS6 (“Treated with less respect than usual”) (average edge weight = −0.033) (Figs. 3 and 4, Supplementary Tables S1 and S2).

**Fig. 5:**
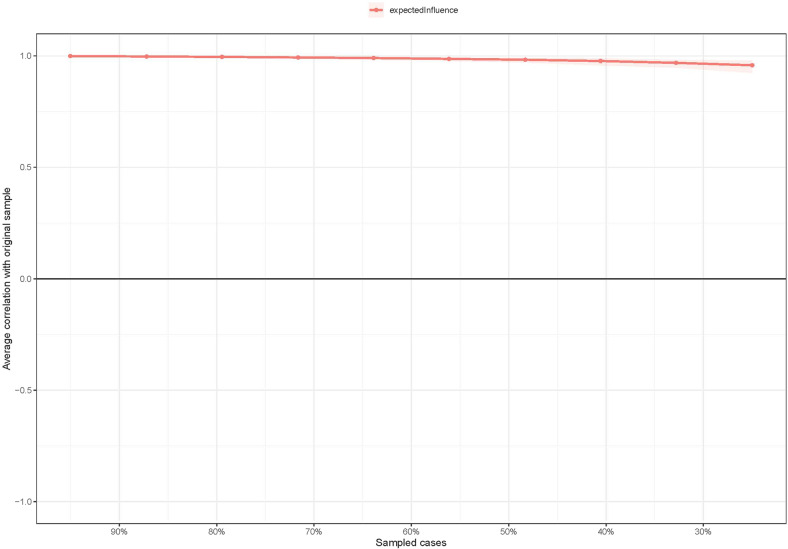
The stability of centrality indice using case-dropping bootstrap. The *x*-axis represents the percentage of cases of the original sample used at each step. The y-axis represents the average of correlations between the centrality indices in the original network and the centrality indices from the re-estimated networks after excluding increasing percentages of cases. The red line indicates the Expected Influence.

The EI centrality had an excellent level of stability (i.e., CS-coefficient = 0.672). Furthermore, results of bootstrapped differences tests for edge weights show that most comparisons between edge weights were statistically significant (Fig. 5, Supplementary Fig. S1).

## Discussion

This study was the first to examine COVID-19 acceptance rates as well as its links with overall mental illness stigma concerns within a large depressed patient sample. In addition, we employed network analysis to identify the central or most influential individual nodes within the mental illness stigma-vaccine acceptance network model of the sample and specific bridge nodes linking mental illness stigma with vaccine acceptance.

Regarding the vaccination acceptance rate, 53.7% of depressed patients in this research expressed willingness to receive a COVID-19 vaccine, a rate that was considerably lower than percentages in non-clinical adult samples which have ranged from 69% to 80% in countries such as England, Denmark, the United States, Australia, and France [34, 38, 60, 61]. The current acceptance rate was also substantially lower than estimates for the general Chinese adult population (88.6%) [34] and Chinese adolescents (75.6%) [35] as well as the acceptance rate in an Australian study of people with schizophrenia queried about the H1N1 influenza vaccines (74%) [62]. A number of factors could have contributed to the comparatively low rate of COVID-19 vaccine acceptance in depressed patients. Patients with major psychiatric disorders including depression may have inadequate access to accurate information about COVID-19 vaccinations [63] due, in part, to symptoms of their disorder and impaired cognitive abilities [64]. In addition, some patients may be concerned about potential side-effects of COVID-19 vaccines on their symptoms and medications [65], a concern that is somewhat founded because there have been no specific vaccine guidelines for people with severe mental illnesses including depression [65].

Regarding the main research focus, higher perceived stigma related to having depression was associated with lower acceptance of COVID-19 vaccines in the sample. Notably, this association remained statistically significant even after the impact of other significant correlates of vaccine acceptance (i.e., depression severity, suicidality, inpatient status, pain severity) had been statistically controlled within a hierarchical multiple regression model. As such, the relationship between experiences of being stigmatized for having depression and vaccine acceptance could not be explained by other patient factors including severity of depression or experiences of suicidality.

This finding dovetails with other evidence indicating the stigma of having a mental illness is a potential barrier to seeking healthcare among people with psychiatric disorders [24, 32]. Because stigma reflects disapproval of “outgroups” that have particular attributes [23], people with a history of depression or other mental illnesses may feel judged, devalued, or dehumanized by others in their social environments, including health professionals with whom they come into contact [25, 26, 28]. Consequently, psychiatric patients who have had frequent encounters with being stigmatized are more prone to viewing contact with the health care system as a threat to their self-worth [30–32] and experience general reluctance in seeking healthcare [33], even when interventions such as vaccinations have no direct bearing on their disorders.

Aside from the unique influence of perceived stigma, factors associated with severity of depressive illness (i.e., experiences of suicidality in the past year, status as an inpatient, severity of current depression and current pain) emerged as significant correlates of reduced vaccine acceptance in our sample. These findings align with conclusions from a recent review of related evidence suggesting that people with severe mental illness have a higher risk for poor COVID-19-related outcomes than do people with less severe mental illness due to several factors including more highly compromised immune systems, increased sleep disturbances, lower socioeconomic status, and more limited access to appropriate care [5]. For clinicians who work with depressed patients, our results underscore severity of illness as an important consideration in identifying depressed patients who are less willing to be vaccinated and selecting interventions that are effective in reducing severe depressive symptomatology [66–68].

Aside from assessing overall correlates of COVID-19 vaccine acceptance, we identified specific mental illness stigma experiences that were most influential within the mental illness stigma-vaccine acceptance network model and those having the strongest links with vaccine acceptance. “Feel others avoid me because of my illness”, “Feel useless”, and “Feel less competent than I did before” emerged as the central or influential nodes in the network model, independent of depression severity. Perceptions of others’ avoidance due to one’s illness as a key central symptom aligns with past evidence indicating loneliness and low social support contribute to higher perceived stigma among patients with mental illness [69, 70]. By definition, stigma highlights particular characteristics that distinguish certain groups from other members of society [23], so mental illnesses such as depression may perpetuate feelings of “otherness” and isolation that depressed persons may feel in their interactions with others.

“Feel useless” (SIS23) and “Feel less competent than I did before” (SIS20) were the other key central symptoms that emerged in the network model. Feelings of uselessness and reduced competence are common to depression; for example, loss of energy or fatigue, anhedonia, diminished worth and impaired functioning are depression criteria that may perpetuate perceptions of uselessness and incompetence [71]. Hence, the impact of perceived “uselessness” and lower competence as central stigma experiences within the current network model may have been at least partly the function of having assessed stigma within a group having higher levels of depressive symptomatology. Further network analyses within stigmatized groups that are not depressed are needed to test the extent to which these facets of stigma are central to depressed samples versus other stigmatized groups.

Notably, however, perceptions of being avoided due to depression, feelings of uselessness, and doubts about personal competence emerged as most influential nodes in the network model for mental illness stigma and COVID-19 acceptance, after controlling for overall depression severity scores. Hence, the occurrence or perception of being avoided or ostracized and of coming to view oneself as useless and less competent may also be important for the onset or persistence of mental illness stigma among depressed patients. Other studies have confirmed the relevance of these experiences to stigma. For example, one review of the literature on public stigma indicated patients with mental illnesses are often viewed as lazy or less competent in making personal decisions related to treatment and finances [72]. Experimental research has found that, compared to job candidates with a history of physical injuries, job candidates with a mental illness history are more likely to be discriminated against, in part, due to diminished expectations of competence [73]. Presumably, external feedback from others and internalization of such messages perpetuate subjective perceptions of being avoided, personal uselessness or incompetence, and other aspects of feeling stigmatized among people with depression [74].

In other analyses, elevations on mental illness stigma items related to social rejection (“Employers/co-workers have discriminated”, “Treated with less respect than usual”) and social isolation (“Sense of inequality in my relationships with others”) were stigma items having the strongest associations with lower COVID-19 vaccine acceptance in the sample, controlling for depression severity. Numerous past studies have found stigmatized groups that experience rejection or discrimination within the healthcare system (i.e., provider discrimination) are more likely to mistrust medical advice, less likely to pursue and comply with preventive health services, and more likely to delay seeking medical care [75–79]. For example, psychiatric patients may be vulnerable to discrimination during the COVID-19 pandemic due to perceptions that they are less able to care for themselves and their own potential difficulties adhering to personal protection strategies such as hand-washing and use of masks [80, 81]. Our bridge node results are also consistent with evidence of significant inverse relations between discrimination or social rejection experiences that occur in daily life and healthcare use among the mentally ill [82, 83]. Findings also converge with premises of identity threat models whereby people who have experienced social rejection or discrimination adopt a disengagement strategy in which they avoid dominant cultural institutions, including healthcare, to cope with fears that they will be stigmatized further [74].

In tandem with the significant unique association between overall elevations in mental illness stigma and reluctance in accepting COVID-19 vaccinations, network analysis findings from this study suggest that interventions to reduce mental illness stigma may be useful in efforts to increase vaccination rates among depressed patients. To date, stigma-related interventions have focused on education (i.e., enhancing knowledge by contrasting myths versus facts of mental illness) versus contact (i.e., equal-status interactions between the public and people in recovery from serious mental illness) [84]. Meta-analyses of this literature have indicated education alone (e.g., more facts about what is an illness) typically has a more limited impact in reducing public stigma related to mental illness [84–87]. Instead, approaches that emphasize indirect contact (e.g., videoclips of patients) or direct contact with patients, either as stand-alone approaches or with an accompanying education component tend to produce better overall outcomes [86]. Aside from the need to communicate the essential humanity of people with depression or other mental illnesses in contact-based interventions that reduce public stigma of mental illness [85], our network model results underscore the possible utility of targeting depressed patient concerns about inequality and others’ avoidance in addition to managing experiences of social rejection in daily life, feelings of uselessness, and competence doubts.

Strengths of this study included its large sample, multi-center study design, and use of network analysis along with traditional analysis strategies. However, its main limitations should also be noted. First, due to the cross-sectional design, causal effects of mental illness stigma, depression severity and other factors on COVID-19 vaccination acceptance could not be demonstrated. Second, possible effects of selection biases cannot be ruled out because random sampling was not used in recruitment. Third, other possible correlates of COVID-19 vaccine acceptance such as psychiatric comorbidities and social support were not assessed to maintain relatively low response burdens on unpaid research volunteers. Fourth, because COVID-19 vaccines were not widely available in China prior to the launch of this study (i.e., October 1, 2020), extensions that examine associations between facets of perceived mental illness stigma and actual COVID-19 vaccine uptake in depressed patients should be a future research focus given that COVID-19 vaccines are now widely available.

In conclusion, slightly less than half of depressed patients assessed in this study reported at least some reluctance in receiving a COVID-19 vaccine. Furthermore, elevations in perceived stigma with having a mental illness and factors reflecting increased severity of depression were related to increased hesitancy in being vaccinated within the sample. As such, mental health professionals should consider these issues in assessments of vaccine-hesitant depressed patients. Relatedly, interventions to reduce stigmatizing attitudes of the general public as well as social inclusion opportunities for patients with heightened stigma concerns should be developed in tandem with traditional interventions to reduce depression severity.

## Supplementary information

Supplementary materials

## Data Availability

The Institutional Review Board (IRB) of Beijing Anding Hospital that approved the study prohibits the authors from making the research data set publicly available. Readers and all interested researchers may contact Dr. Yu-Tao Xiang (Email address: xyutly@gmail.com) for details. Dr. Xiang could apply to the Institutional Review Board (IRB) of Beijing Anding Hospital for the release of the data.
