# Supplementary material for: COVID-19 vaccine acceptance and perceived stigma in patients with depression: a network perspective
Source: Transl Psychiatry. 2022 Oct 4;12:429. doi: 10.1038/s41398-022-02170-y (PMC9530420; doi:10.1038/s41398-022-02170-y)
Supplement: Supplementary file 1 — Supplementary materials [file 41398_2022_2170_MOESM1_ESM.docx]

**Supplementary Material**

Table S1. Means, standard deviations, skewness, and kurtosis

Table S2. Correlation matrix of the COVID-19 vaccination acceptance, PHQ-2 and SIS items

Figure S1. Nonparametric bootstrapped difference test

**Table S1. Means, standard deviations, skewness, and kurtosis**

| Item | M | SD | Skewness | kurtosis |
| --- | --- | --- | --- | --- |
| PHQ-2 total | 5.16 | 1.89 | 0.02 | -1.01 |
| COVID-19 vaccination acceptance | 0.54 | 0.50 | -0.15 | -0.98 |
| SIS1 | 2.37 | 0.79 | -0.07 | -0.56 |
| SIS2 | 1.98 | 0.72 | 0.49 | 0.21 |
| SIS3 | 2.84 | 0.73 | -0.46 | 0.25 |
| SIS4 | 2.64 | 0.80 | -0.30 | -0.31 |
| SIS5 | 2.28 | 0.77 | 0.31 | -0.19 |
| SIS6 | 2.49 | 0.77 | -0.05 | -0.39 |
| SIS7 | 2.20 | 0.78 | 0.31 | -0.25 |
| SIS8 | 2.99 | 0.74 | -0.58 | 0.42 |
| SIS9 | 2.83 | 0.76 | -0.38 | -0.06 |
| SIS10 | 2.87 | 0.74 | -0.43 | 0.11 |
| SIS11 | 2.56 | 0.82 | -0.06 | -0.53 |
| SIS12 | 2.13 | 0.79 | 0.37 | -0.24 |
| SIS13 | 2.16 | 0.80 | 0.29 | -0.39 |
| SIS14 | 2.06 | 0.74 | 0.40 | 0 |
| SIS15 | 2.68 | 0.77 | -0.26 | -0.26 |
| SIS16 | 2.11 | 0.78 | 0.35 | -0.25 |
| SIS17 | 2.12 | 0.81 | 0.23 | -0.60 |
| SIS18 | 2.69 | 0.77 | -0.42 | -0.08 |
| SIS19 | 2.28 | 0.76 | 0.29 | -0.18 |
| SIS20 | 2.02 | 0.75 | 0.43 | -0.06 |
| SIS21 | 2.44 | 0.77 | 0.05 | -0.39 |
| SIS22 | 2.54 | 0.78 | -0.07 | -0.40 |
| SIS23 | 2.12 | 0.79 | 0.34 | -0.31 |
| SIS24 | 2.50 | 0.80 | -0.19 | -0.48 |

**Table S2. Correlation matrix of the COVID-19 vaccination acceptance, PHQ-2 and SIS items**

|  | SIS1 | SIS2 | SIS3 | SIS4 | SIS5 | SIS6 | SIS7 | SIS8 | SIS9 | SIS10 | SIS11 | SIS12 | SIS13 | SIS14 | SIS15 | SIS16 | SIS17 | SIS18 | SIS19 | SIS20 | SIS21 | SIS22 | SIS23 | SIS24 | COV | PHQ-2 total |
| --- | --- | --- | --- | --- | --- | --- | --- | --- | --- | --- | --- | --- | --- | --- | --- | --- | --- | --- | --- | --- | --- | --- | --- | --- | --- | --- |
| SIS1 | 0 |  |  |  |  |  |  |  |  |  |  |  |  |  |  |  |  |  |  |  |  |  |  |  |  |  |
| SIS2 | 0.134 | 0 |  |  |  |  |  |  |  |  |  |  |  |  |  |  |  |  |  |  |  |  |  |  |  |  |
| SIS3 | 0.055 | 0 | 0 |  |  |  |  |  |  |  |  |  |  |  |  |  |  |  |  |  |  |  |  |  |  |  |
| SIS4 | 0.227 | 0.023 | 0.238 | 0 |  |  |  |  |  |  |  |  |  |  |  |  |  |  |  |  |  |  |  |  |  |  |
| SIS5 | 0.006 | 0.167 | 0.056 | 0.082 | 0 |  |  |  |  |  |  |  |  |  |  |  |  |  |  |  |  |  |  |  |  |  |
| SIS6 | 0.019 | 0.001 | 0.067 | 0.154 | 0.255 | 0 |  |  |  |  |  |  |  |  |  |  |  |  |  |  |  |  |  |  |  |  |
| SIS7 | 0.003 | 0.110 | 0 | 0 | 0.032 | 0.113 | 0 |  |  |  |  |  |  |  |  |  |  |  |  |  |  |  |  |  |  |  |
| SIS8 | 0.035 | 0 | 0.031 | 0.063 | 0 | 0.053 | 0 | 0 |  |  |  |  |  |  |  |  |  |  |  |  |  |  |  |  |  |  |
| SIS9 | 0.007 | 0 | 0.042 | 0.028 | 0 | 0.060 | 0.010 | 0.224 | 0 |  |  |  |  |  |  |  |  |  |  |  |  |  |  |  |  |  |
| SIS10 | 0 | 0 | 0.120 | 0 | 0 | 0 | 0 | 0.155 | 0.368 | 0 |  |  |  |  |  |  |  |  |  |  |  |  |  |  |  |  |
| SIS11 | 0 | 0 | 0.092 | 0 | 0.025 | 0.009 | 0.050 | 0.022 | 0.112 | 0.112 | 0 |  |  |  |  |  |  |  |  |  |  |  |  |  |  |  |
| SIS12 | 0 | 0 | 0 | 0 | 0 | 0 | 0 | 0 | 0.008 | 0 | 0.020 | 0 |  |  |  |  |  |  |  |  |  |  |  |  |  |  |
| SIS13 | 0 | 0 | 0 | 0 | 0 | 0 | 0 | 0 | 0 | 0 | 0.020 | 0.320 | 0 |  |  |  |  |  |  |  |  |  |  |  |  |  |
| SIS14 | 0 | 0 | 0 | 0 | 0 | 0 | 0 | -0.023 | 0 | 0 | 0 | 0.354 | 0.333 | 0 |  |  |  |  |  |  |  |  |  |  |  |  |
| SIS15 | 0.026 | 0 | 0.040 | 0.025 | 0 | 0.064 | 0 | 0.025 | 0.225 | 0.108 | 0.060 | 0.026 | 0.024 | 0.033 | 0 |  |  |  |  |  |  |  |  |  |  |  |
| SIS16 | 0.005 | 0 | 0 | 0 | 0 | 0.016 | 0.024 | 0 | 0 | 0 | 0 | 0 | 0.032 | 0.112 | 0.0124 | 0 |  |  |  |  |  |  |  |  |  |  |
| SIS17 | 0 | 0 | 0 | 0 | 0.001 | 0.055 | 0.107 | 0 | 0 | 0 | 0.034 | 0.044 | 0.018 | 0 | 0.015 | 0.206 | 0 |  |  |  |  |  |  |  |  |  |
| SIS18 | 0.001 | 0 | 0.093 | 0.033 | 0 | 0.043 | 0 | 0.073 | 0.057 | 0.099 | 0.037 | 0 | 0 | 0 | 0.137 | 0 | 0.099 | 0 |  |  |  |  |  |  |  |  |
| SIS19 | 0.001 | 0.020 | 0 | 0 | 0 | 0 | 0.045 | 0 | 0 | 0 | 0.210 | 0.009 | 0 | 0.048 | 0.017 | 0.041 | 0.052 | 0.0376 | 0 |  |  |  |  |  |  |  |
| SIS20 | 0 | 0.148 | -0.013 | 0 | 0.191 | 0.008 | 0.166 | -0.025 | 0 | -0.009 | 0 | 0.025 | 0.018 | 0.008 | 0 | 0.042 | 0.101 | 0 | 0.053 | 0 |  |  |  |  |  |  |
| SIS21 | 0.043 | 0.032 | 0 | 0.041 | 0.055 | 0.058 | 0 | 0 | 0 | 0.040 | 0.020 | 0.013 | 0.093 | 0 | 0 | 0.048 | 0 | 0.125 | 0.002 | 0.047 | 0 |  |  |  |  |  |
| SIS22 | 0 | 0 | 0 | 0.017 | 0 | 0 | 0 | 0.021 | 0.037 | 0.025 | 0.016 | 0.021 | 0.014 | 0 | 0.116 | 0 | 0 | 0.169 | 0.072 | 0 | 0.275 | 0 |  |  |  |  |
| SIS23 | 0.006 | 0.046 | 0 | 0 | 0 | 0 | 0.099 | 0 | 0 | 0 | 0.026 | 0.014 | 0.109 | 0 | 0 | 0.11 | 0.121 | 0 | 0.161 | 0.225 | 0.044 | 0.07 | 0 |  |  |  |
| SIS24 | 0 | 0 | 0 | 0.111 | 0.039 | 0.081 | 0 | 0 | 0 | 0 | 0 | 0.006 | 0.048 | 0 | 0.090 | 0.01 | 0 | 0.103 | 0.044 | 0.008 | 0.04 | 0.065 | 0.09 | 0 |  |  |
| COV | 0 | 0 | -0.046 | 0 | -0.006 | -0.033 | 0 | -0.032 | 0 | -0.00014 | -0.021 | 0 | 0 | 0 | 0 | 0 | 0 | 0 | -0.013 | 0 | 0 | 0 | 0 | -0.003 | 0 |  |
| PHQ-2 total | 0 | 0.041 | -0.031 | -0.018 | 0 | 0 | 0.108 | 0 | 0 | 0 | 0.0003 | 0 | 0 | 0 | 0 | 0 | 0.159 | 0 | 0 | 0.139 | 0 | 0 | 0.064 | 0 | 0 | 0 |

**Figure S1. Nonparametric bootstrapped difference test**


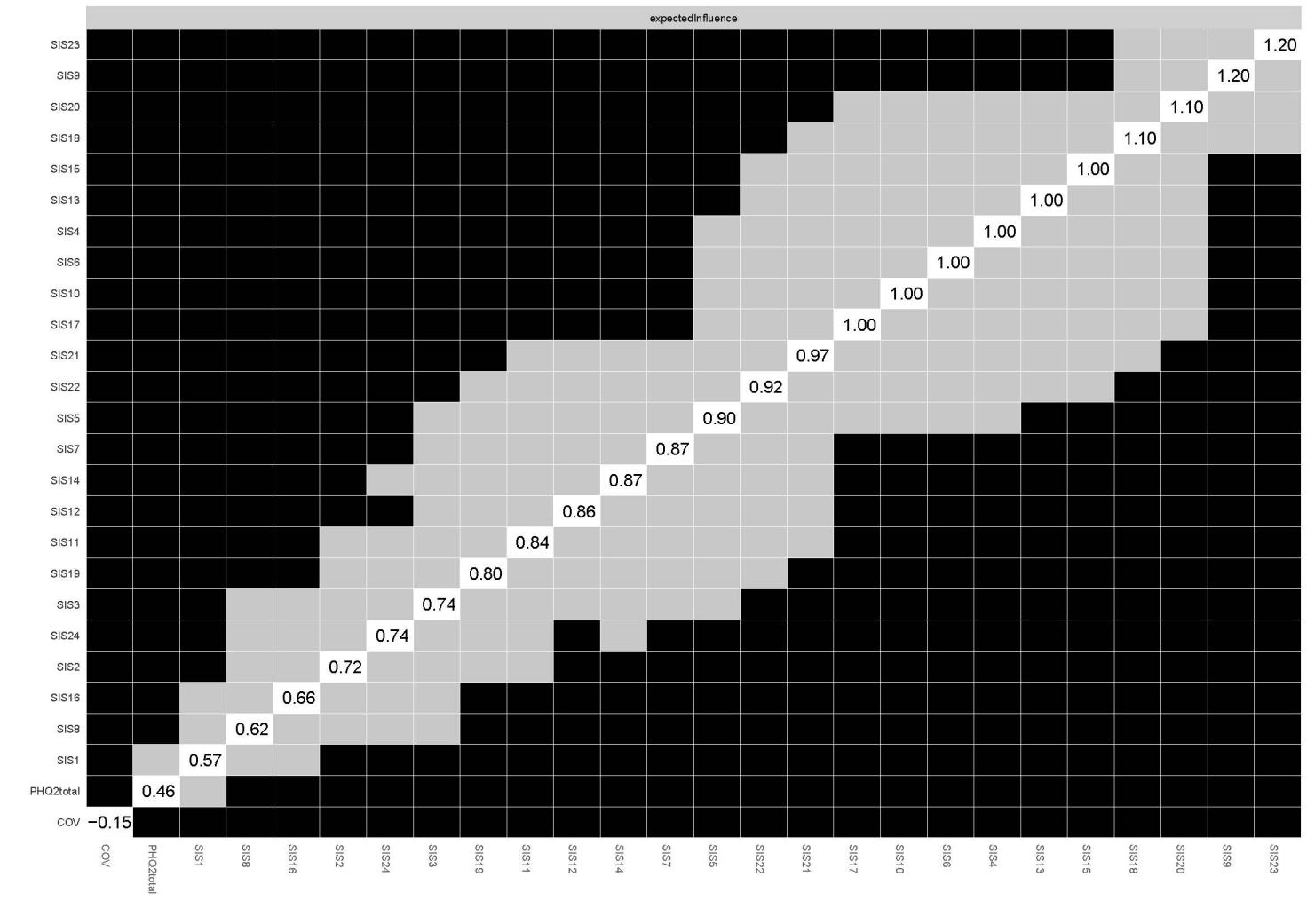


Figure S1. Nonparametric bootstrapped difference test for expected influence. Bootstrapped difference tests between edge weights in the network. Gray boxes indicate edges that do not significantly differ from one-another. Black boxes represent edges with significant difference from one another. Blue boxes in the edge-weight plot indicate positive correlations.
